# Gender-specific determinants of Zai technology use intensity for improved soil water management in the drylands of Upper Eastern Kenya

**DOI:** 10.1016/j.heliyon.2021.e07217

**Published:** 2021-06-04

**Authors:** Amos Mwenda Ndeke, Jayne Njeri Mugwe, Hezron Mogaka, George Nyabuga, Milka Kiboi, Felix Ngetich, Monicah Mucheru-Muna, Isaya Sijali, Daniel Mugendi

**Affiliations:** a Department of Agricultural Economics and Extension, University of Embu, PO Box 6-60100, Embu, Kenya; b Department of Agricultural Science and Technology, Kenyatta University, PO Box 43844-00100, Nairobi, Kenya; c School of Journalism and Mass Communication, University of Nairobi, PO Box 30197-00100, Nairobi, Kenya; d Department of Land and Water Management, University of Embu, PO Box 6-60100, Embu, Kenya; e Department of Environmental Sciences and Education, Kenyatta University, PO Box 43844-00100, Nairobi, Kenya; f Food Crops Research Centre-Kabete, Kenya Agricultural and Livestock Research Organization (KALRO), PO Box 14733-00800, Nairobi, Kenya

**Keywords:** Male-headed households, Female-headed households, Choice, Heckman-two-step selection model

## Abstract

Degraded landscapes and soil water stress are long-standing problems to smallholder agriculture in the drylands. Despite the important roles of zai technology in restoring degraded landscapes and improving agricultural productivity, the technology is yet to be adopted to its fullest extent. This can be attributed to gender-linked disparities in agricultural technology utilization. The study, therefore, sought to determine gender-specific determinants of zai technology choice and use-intensity. A multistage sampling technique was employed in randomly selecting 133 female-headed households and 267 male-headed households in Tharaka South sub-county. Quantitative data were collected in a cross-sectional survey using an interviewer-administered questionnaire. Using sex-disaggregated data, Chi-square and t-test statistic were employed to test the statistical significance of dummy and mean value of continuous variables, respectively. Gender specific determinants of zai technology choice and use-intensity were determined using the Heckman-two-step econometric model. The results revealed that, more women farmers (44%) were using zai technology as compared to men (38%). Among women farmers, total cultivated land, access to animal-drawn farm implements, and group membership had an influence on zai technology choice. For men, total cultivated land, group membership and access to extension services positively influenced choice of zai technology. With regard to zai technology use-intensity, total land cultivated, livestock densities, group membership and frequency of trainings on soil and water management were important determinants among women farmers. For men, zai technology use-intensity was determined by total cultivated land and farmers’ perceptions on soil erosion. We recommend that, gender-sensitive farm-level policies oriented towards farmer socioeconomic profiles are important deliberations towards choice and intense application of soil and water conservation strategies such as the zai technology.

## 1 Introduction

Climate change exacerbates food insecurity as variations in agroclimatic conditions impinge sustainable food production, especially in the dryland systems (IPCC, 2014; Farooq and Siddique, 2017). As was noted by White et al. (2002), arid and semi-arid lands cover about 40% of land surface globally, but most extensive in Africa (13×l0^6^ km^2^). Correspondingly, in sub-Sahara Africa (SSA), there is a high incidence of food insecurity where rain-fed subsistence agriculture remains a predominant livelihood strategy for most people residing in the drylands (Shahid and Al-Shankiti, 2013; Barbier and Hochard, 2018). These regions experience erratic rainfall, recurrent dry spells, increasing temperatures, and infertile lands characterized by; diminishing organic matter and reduced biological activity, and this poses limitations for intensifying agricultural productivity (Bradford et al., 2017, 2019; Issahaku and Abdul-Rahaman, 2019). Upper Eastern Kenya faces similar challenges of soil moisture stress, declining soil fertility, and reduced agricultural yields promoting various research and development efforts on soil and water conservation (Mucheru-Muna et al., 2010; Ngetich et al., 2014; Kimaru-Muchai et al., 2020). In response to these challenges, smallholder farmers usually apply various conservation strategies, but often at lower rates than the recommended (Mugwe et al., 2009; Kiboi et al., 2017; Mwaura et al., 2021). This aggravates production volatility heightening the food crisis in the rural economies (Mganga et al., 2015; Rojas et al., 2016; Sinyolo, 2020).

The growing risk of vulnerability to climate shocks is not gender-neutral (Djoudi and Brockhau 2011; Beuchelt & Badstue et al., 2013). Women farmers face different challenges in utilizing agricultural innovations to avert climate-related risks when compared to their male counterparts (Diouf et al., 2019; Rola-Rubzen et al., 2020). Furthermore, gender inequalities and lack of attention to men and women's specific preferences and needs is associated with low use of agricultural innovations (Huyer, 2016; Kawarazuka et al., 2018; Rola-Rubzen et al., 2020). The disparities exist in form of land tenure insecurities, to which women farmers are underprivileged in use and decision making; gender differences in access to education and extension trainings; rationing out of credit markets; greater difficulties in access and control to assets including, livestock and farm implements and machinery; limited access to education and agricultural training, and other social and cultural forms of inequalities linked to social perceptions on differentiated roles for men and women (Njuki et al., 2011; Quisumbing et al., 2014; Doss et al., 2018). Moreover, patriarchal systems are oppressive to women, perhaps not allowing women farmers to participate more effectively in decision-making (Sultana, 2012; Colfer et al., 2015; Mukoni, 2018). Consequently, the inequalities have implications for technology use and pose a significant drawback to the effective utilization of agricultural innovations (Rola-Rubzen et al., 2020). In the pursuit for women's empowerment in agriculture, aligning the design and implementation of agricultural technologies to specific gender preferences is imperative.

The zai technology remains a dependable choice for improving soil water conservation in the drylands (Danso-Abbeam et al., 2019; Kimaru-Muchai et al., 2020). Precisely, as an effort to bridge intraseasonal dry spells, development agencies in the drylands of upper Eastern Kenya introduced and incessantly promote the use of zai technology (Kimaru-Muchai et al., 2020). Zai technology is recommended for drier agro-ecological zones receiving 300–800 mm annual rainfall (Roose et al., 1999), hence best-fitting the region. Farmers developed zai technology "small planting, water harvesting basins filled with manure, compost or dry biomass" in the early 1960s (Partey et al., 2018). Ever since, the approach has been extensively improved, promoted, and adopted (Sawadogo et al., 2001; Danjuma and Mohammed, 2015; Nyamekye et al., 2018) for restoration and rehabilitation of completely denuded, encrusted degraded land and in landscapes where runoff is prevalent (Roose et al., 1999).

In the restoration practice, runoff collection basins of dimensions 20–40 cm diameter and 10–15 cm deep are implemented early before the onset of rains (Roose et al., 1999). The pit size is subject to variations; deeper pits in shallow horizons and shallow pits on the watertight soils (Slingerland and Stork, 2000). For example, in Kenya, most farmers observe 60 cm × 60 cm × 60 cm dimensions in width, length and height when executing zai (Peter and Itabari, 2014). On average, with a spacing of 60–80 cm apart, 8000 pits fit ha^−1^ (Fatondji et al., 2006; Kimaru-Muchai et al., 2020), and are applied in alternating rows to increase runoff collection. In most cases, on average, farmers incorporate zai pits with about 2 Mg ha^−1^ of well decomposed manure or crop residues (Roose et al., 1999; Mwangi, 2020). Moreover, some farmers incorporate mineral fertilizer in the pits (Kimaru-Muchai et al., 2020). The addition of organic matter improves runoff water infiltration, thus creating deep moisture pockets in the planting hole, protected from quick evaporation (Danso-Abbeam et al., 2019). The incorporation of manure and other organic residues also helps in maintaining soil structure. Decomposition of organic matter by soil organisms enrich the soils and runoff water with nutrients (Roose et al., 1999). Subject to rainfall and soil fertility conditions, on average, well-executed zai pits can lead to about 750 kg ha^−1^ of grain yields and about 3 Mg ha^−1^ of crop residue for mulching and livestock feed (Fatondji et al., 2006). Also, zai harvests 25% of surface runoff from 5 times its area (Malesu, 2006) and increases soil water holding capacity by over 500% (Danjuma and Mohammed, 2015). Furthermore, water conservation structures achieve dual purposes of increased spatial extent and duration of plant-available moisture and controlling soil erosion by trapping and altering sediment distribution (Nichols et al., 2021). Conversely, the use of the technology among other soil and water management technologies has stagnated over time in spite of its diffusion (Mugwe et al., 2019; Kimaru-Muchai et al., 2020).

Recently, several studies that consider agricultural technology use have reported imperfect information and institutions among other demographic and socioeconomic characteristics to be constraining factors to utilization of agrarian technologies (Mango et al., 2017; Wekesa et al., 2018; Thinda et al., 2020). On the other hand, empirical evidence has proven that gender inequalities exist in utilization of agricultural technologies owing to inadequate access to key productive assets, education and relevant training among other fairly obvious and largely overlooked technical constraints (Ndiritu et al., 2011; Meinzen-Dick et al., 2019; Rola-Rubzen et al., 2020). Integrating gender in understanding the synergies between factors underlying choice and use-intensity of the zai technology is crucial in crafting, diffusion and intensification. Thus, in this study, we assessed gender-specific determinants of choice and use-intensity of the zai technology using household-level data.

## 2 Materials and methods

### 2.1 Study area

**Figure 1:**
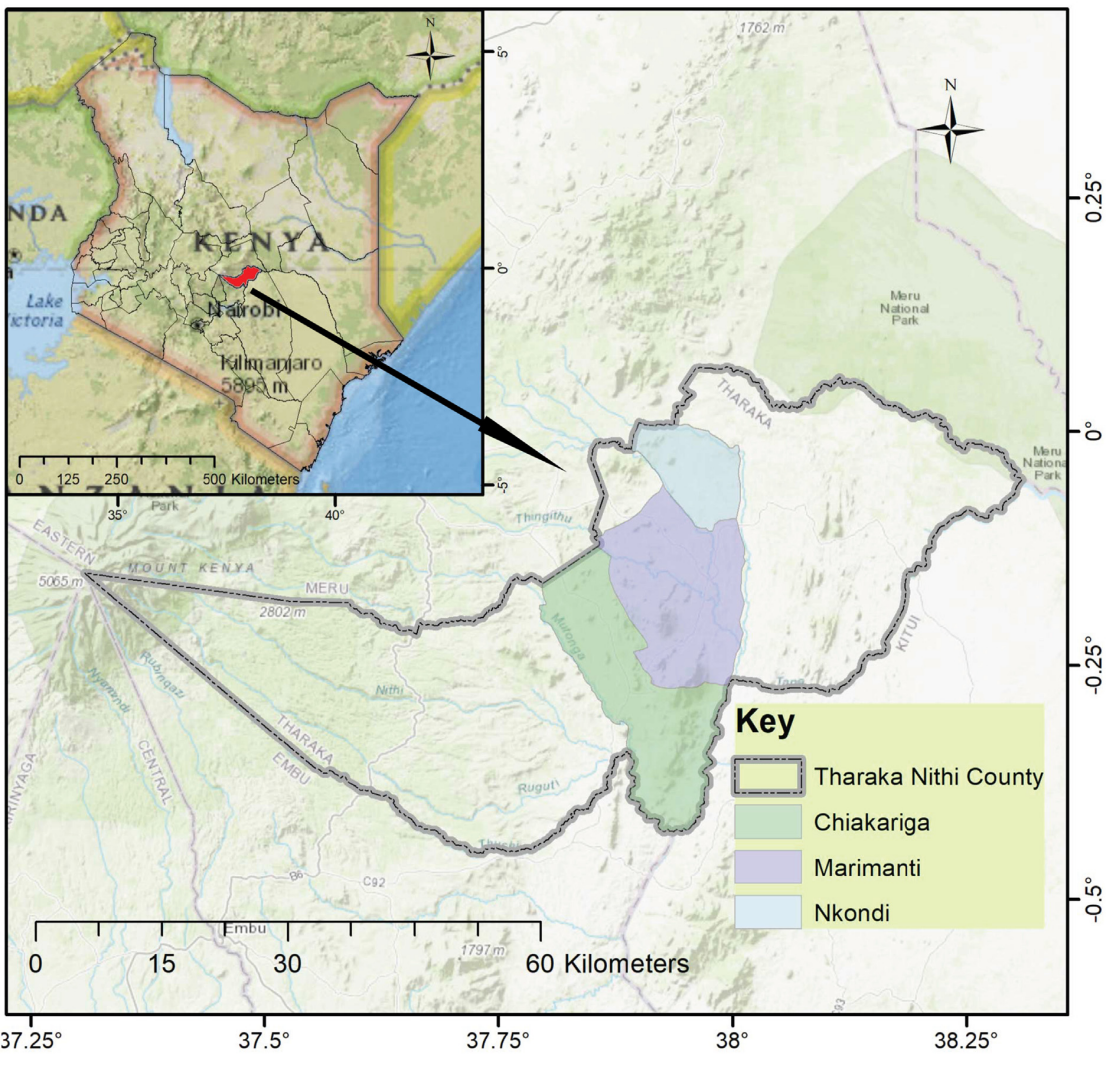
Map of the study area.

The study was conducted in Tharaka Nithi County, Kenya, covering three wards in Tharaka South sub-county: Chiakariga, Marimanti, and Nkondi (Figure 1). The sub-county covers about 637 km^2^ with a population of 75,250 persons, and a population count of approximately 118 persons per square kilometre (Kenya National Bureau of Statistics, 2019). The Agro-Ecological Zones (AEZs) covering the area range from the wetter Lower Midland (LM)4 to the drier Intermediate Lowland (IL)6 (Jaetzold et al., 2007). The area receives bi-modal rainfall: March–May "long rains" and October–December "short rains" (Jaetzold al., 2007; Recha et al., 2012). The annual rainfall amount ranges from 1100 mm in the LM4 to less than 800 mm in the IL6. Farmers in the region prefer the October–December season for its reliability and accurate predictability. The annual temperature ranges from 21 to 25 °C (Smucker and Wisner, 2008). Shallow, highly weathered, and leached Ferrasols are the main soils in Tharaka South sub-county (Jaetzold et al., 2007). As a semiarid sub-county, rainfall is highly variable, affecting the community livelihood strategies, which is primarily agro-pastoralism (Smucker and Wisner, 2008; Recha et al., 2012). The sub-county's erratic rainfall has contributed to wide variability in crop and livestock production, escalating poverty levels and overdependence on relief from government and development agencies (Muriu Ng'ang'a et al., 2017; Kimaru-Muchai et al., 2020). Ongoing development efforts in the area along with devolution target diversification of livelihood options that are responsive to climate change. The choice of the sub-county was guided by earlier research efforts in the area and the understanding that being a semiarid area, livelihood options are limited and vulnerability levels differ across gender and households.

### 2.2 Sample size, sampling strategy, and data collection

Sampling units were drawn using multistage sampling procedures. Tharaka South Sub-county was pre-defined because farmers in that region practice zai technology among other soil and water conservation innovations. In the second stage, all the three wards (Chiakariga, Marimanti and Nkondi) in the Tharaka South sub-county were selected using a sampling framework. In the third stage; at ward level, sample households were randomly selected. A list of 4,000 farmers was obtained from Tharaka South Sub-county agricultural office. The basic element in the sampling frame was the farm household. A probability proportional to size sampling technique was employed to determine the number of households sampled per ward (Table 1). A sample of 400 farming households was randomly selected. Random numbers were generated to reduce the chances of sample selection bias.

**Table 1:** Sampled wards and their number of respondents.

Ward	Male-headed households	Female-headed households	Total
Chiakariga	117	67	184
Marimanti	97	38	135
Nkondi	53	28	81
Total	267	133	400

The sample size was determined using Cochran (2007) formula (Eq (1)) as given below:

(1) $$n=\frac{Z^{2\;}pq}{d^{2}}\approx \frac{1.96^{2\;}*\left(0.5\right)*\left(1-0.5\right)}{0.049^{2}}\approx 400$$

Where n = sample size, Z = 1.96 the standard normal deviate at the required confidence level, p = (0.5) the proportion in the target population estimated to have the characteristic under observation, q = 1-p = 0.5 = the proportion of the population without the characteristics being measured d = 0.049 = the desired level of precision. In total, 400 farmers were sampled.

We used semi-structured questionnaires with modules on-farm and farmer's socioeconomic characteristics and institutional factors to collect quantitative data at the household level in a cross-sectional survey. We programmed the questionnaire into an electronic format using Open Data Kit (ODK) software and sufficiently pre-tested for reliability and validity and corrected for errors. Trained enumerators were used in administering the questionnaires.

### 2.3 Data processing and statistical analysis

Livestock densities were determined for each unit following Musafiri et al. (2020). For every cow, sheep, goat, and chicken, Total Livestock Unit (TLU) of 0.7, 0.1,0.1, and 0.01, was assigned respectively. Area of land was converted to hectares. Secondly, data was cleaned, organized in Microsoft Excel, and analysed using STATA and SPSS softwares'. The analyses disaggregated the results by sex of the household head based on key indicators of the study. Chi-square and t-test were employed to test statistical relationships for categorical and continuous variables respectively. Comparisons were made between zai technology users and non-users in male-headed households and female-headed households. Heckman's selection model was employed in estimating the determinants of zai technology choice and use-intensity in Upper Eastern Kenya's drylands.

### 2.4 Conceptual and methodological framework

#### 2.4.1 Theoretical framework

The study followed random utility maximization theory, which postulates that, a rational farmer will choose a given innovation or a bundle of innovations if the benefits derived from their choice exceed the benefits derived from not choosing (Feder and Umali, 1993). The utility (*u)* that an individual (*m)* gains from utilizing *(n)* soil and water conservation technologies can be defined by Eq. (2), where (*v)* is the utility determinants and *(Ɛ)*is the error term*.*

(2) $$u_{mn}=v_{mn}+\varepsilon_{mn}$$

We assumed that (*u)* depends on individual preferences from a package of *(n)* soil and water management alternatives Cascetta (2009). Thus, the utility function can further be expressed as Eq. (3).

(3) $$u_{mn}=V\left(x_{m},\;z_{n}\right)$$

x_*m*_ is the soil and water conservation strategy, and z_*n*_ are farmers' desired technology-specific attributes and farmer characteristics.

A farmer with the intention of maximizing present farm productivity through increased soil and water conservation will select preferred strategy among a set of *(n)* soil and water conservation innovations. The choice of innovation *(n)* is dependent on expected higher benefits when compared to other innovations (q); if $u_{n}$ >_*.*_ $u_{q}$_._ Among other factors, specific characteristics of an innovation influence levels of satisfaction an individual derives from utilizing an innovation. Chances that an individual (m) will choose innovation (n) from a set of selected innovations (q) could be defined by Eq. (4).

(4) $$p\left[\left(v_{n}-\;v_{q}\right)>u\right]+\varepsilon$$

Variations in choice are accounted for by a random element *(Ɛ)*, included in the utility function.

#### 2.4.2 The analytical framework: Heckman's two-step procedure

We employed the Heckman's two step selection model to obtain unbiased estimates at the second stage of decision making. In the Heckman's selection model, we presumed that, sample selection bias existed necessitating unbiased estimation in the second stage (use-intensity) (Jaleta et al., 2013; Lambrecht and Vanlauwe, 2014; Rabbi et al., 2019). Furthermore, when employing the Heckman's selection model, the assumption is that choice and use-intensity are not determined with exactly a similar set of dependent variables. In this study, frequency of training and farmers' perceptions of soil fertility were the identifier variables that only influenced the first stage (probability of choice) but not the second-stage (use-intensity) of zai technology.

A two-step estimation procedure was followed. In the first step, we estimated the probability of zai choice and obtained the Inverse Mill's Ratio (IMR). The IMR was incorporated in estimating the second step as a remedy for sample selection bias. Heckman's model is anchored on two latent variables (Heckman, 1979). The first step expressed as a hypothetical construct, $Z_{i}^{*}$, representing the choice of zai technology in our study, and hinges on a set of independent variables, $W_{i}$, as given in Eq. (5).

(5) $$Z_{i}^{*}=W_{i}^{\prime }\alpha +\varepsilon_{i}$$

Where, α denotes a k-vector of the independent variables, and $\varepsilon_{i}$ represents the error term.

Hypothetical variable ($Z_{i}^{*}$) is not observed, however, we observe a dichotomous variable $\left(Z_{i}\right)$ whether a farmer was using zai technology or not. Then, the binary variable is given in Eq. (6).

(6) $$Z_{i}=\left\{ \begin{array}{l} 1,\;if\;Z_{i}^{*}>0\\ 0,otherwise \end{array}\right.$$

The second equation is linear representing the use-intensity $\;\left(Y_{i}\right)$, and is given by Eq. (7).

(7) $$Y_{i}=X_{i}^{\prime }\theta +u_{i}$$

Where, θ is a k-vector of the explanatory variables, and $u_{i}$ is the error term.

The error terms $\varepsilon_{i}$ (selection equation) and $u_{i}$ (outcome equation) are independent of α and θ.

The use-intensity $Y_{i}$ is observed when a farmer is using zai technology $\;\left(Z_{i}=1\right)$, prompting inconsistent and biased parameter estimates using Ordinary Least Square (OLS). To correct for the inconsistencies in parameter estimates, the following conditional regression function is used (Eq. (8)):

(8) $$E=\left(\frac{Y_{i}}{Z_{i}}>0\right)=X_{i}^{\prime }\theta +\theta_{\lambda }+\lambda_{i}$$

Where *λ*i is the IMR and given as (Eq. (9)):

(9) $$\lambda_{i}=\frac{\phi W_{i}^{\prime }\alpha }{\phi W_{i}^{\prime }\alpha }$$

Where ϕ is the standard normal, probability density function and ϕ represents the cumulative distribution function for a standard random variable. Lambda is unknown, nevertheless, the variables α can be assessed in a probit model with regard to the observed binary outcome $\left(Z_{i}\right)$.

In estimating the second stage, IMR, $\lambda_{i}=\frac{\phi W_{i}^{\prime }\alpha }{\phi W_{i}^{\prime }\alpha }$ is interleaved into outcome equation as independent variable and given as (Eq. (10)).

(10) $$Y_{i}=X_{i}^{\prime }\theta +\theta_{\lambda }\lambda_{i}+u_{i}$$

This gives rise to self-selection bias when θ is nonzero. To avoid self-selection bias and obtain consistent estimators, the model parameters were estimated using the maximum likelihood criterion.

#### 2.4.3 Empirical model specification

In Heckman's selection model, the first step dependent variable was dummy in nature (whether a farmer was using zai technology or not), and was explained using a set of independent variables, namely age, education, household size, off-farm income, total land cultivated, land ownership, access to farm implements, livestock densities, perceptions on soil fertility and soil erosion, farmer training, group membership, access to relief, frequency of training, the number of groups, household head received credit, access to labour, distance to main market and frequency of extension services. The algebraic representation of Heckman's probit selection model was given in Eq. (11).

(11) $$Z_{i}=\alpha X_{i}+\ldots +\alpha X_{n}+\varepsilon$$

Where:

$Z_{i}$ = the decision of the i^th^ farmer to use zai technology.
$X_{i}$ = the vector of independent variables of probability using zai technology by the i^th^ farmer.
α = the vector of the parameter estimates of the explained variables hypothesized to effect the chances of i^th^ farmer choosing zai technology.

In Heckman's outcome model, the dependent variable was continuous (proportion of cultivated land under zai technology). It was also explained using a set of relevant independent variables, namely age, education, household size, off-farm income, total land cultivated, land ownership, access to farm implements, livestock densities, farmer perceptions on soil erosion, access to training, group membership, access to relief, number of groups, household received credit, access to labour, distance to main market and frequency of extension (Eq. (12)):

(12) $$Y_{i}=\theta X_{i}+\ldots +\theta X_{n}+u_{i}$$

Where:

$Y_{i}$ = area of land allocated for zai technology/Total area of land cultivated
$X_{i}$ = the vector of independent variables of zai technology by the i^th^ farmer use-intensity
θ = the vector of the parameter estimates of the independent variables conjectured to effect the outcome stage.

#### 2.4.4 Model diagnostics

We conducted preliminary diagnostics for statistical problems of multicollinearity. Inter-correlation among dependent variables was tested using the Variance Inflation Factor (VIF). The VIF values obtained were below 10, hence the conclusion that, their existed weak inter-association among the explanatory variables. To validate Heckman's 2-stage selection model viability, golden standards in applying the model were observed. Inverse Mills Ratio (Lambda), a function of the correlation coefficient between first and second stage error terms (rho) that accounts for potential sample selection bias was significant; an indication that sample selection bias was resolved for (Wooldridge, 2010; Certo et al., 2016). We, therefore, concluded that Heckman 2-stage model was sufficient in determining zai technology choice and use-intensity from the sample.

#### 2.4.5 Description of dependent and independent variables

Choice of variables was guided by relevant theories and past studies (Feder et al., 1985; Chianu and Tsujii, 2004; Belachew et al., 2020). However, some variables were selected with regard to theorized relationship with the explained variable. Studies included in the choice of variables demonstrate that farm characteristics and farmer attributes mostly influenced choice and use-intensity of agricultural innovations (Kassie et al., 2014; Mango et al., 2017; Thinda et al., 2020). The influence of these variables was tested in the empirical model.

##### 2.4.5.1 Dependent variable

The first stage dependent variable was a dummy variable (whether a farmer chose to use zai technology or not). It takes the value of 1 if yes and 0 otherwise. The second stage dependent variable was a continuous variable and defined as the proportion of cultivated land (ha) dedicated to zai technology. Past studies have conceptualized intensification as the area of land in hectares planted with improved seeds, fertilizer application rate per acre and number of technologies adopted (Feder et al., 1985; Nkonya et al., 1997; Mensah-Bonsu et al., 2017). Additionally, other studies conceptualize use-intensity as the amount of land under a technology (Nchinda et al., 2010; Asfaw et al., 2011).

##### 2.4.5.2 Independent variables

This information could be found in the supplementary materials (see Table 2).

**Table 2:** Summary of descriptions and units of measurement of hypothesized variables.

Variable	Variable description and measurement	Expected sign
**Dependent variables**		
Zai technology choice	Household head decision to use zai technology is a dummy variable: 1 = Yes; 0 = Otherwise	
Zai use-intensity	Proportion of total cultivated land allocated to zai technology in hectares (continuous)	
**Independent variables**		
HHAGE	Age of the household head was measured in years (continuous)	-
HHEDUC	Education of the household head was measured in years of decision making (continuous)	+/-
HHSIZE	Household size was measured in number (continuous)	+
EXTENSION	Access to extension services is a binary variable: 1 = Received extension; 0 = otherwise	+
LIVSTCK	Livestock densities was measured in number (continuous)	+
PERCSOILERSN	Perception on soil erosion is a dummy variable:1 = Not severe; 2 = Moderate; 3 = Very severe	+
FAMEXP	Household head farming experience is a continuous variable measured in years	+
MKTDST	Distance in walking to the nearest input/output market (continuous)	-
LAND	Total land cultivated is a continuous variable measured in hectares	+/-
CREDIT	Access to credit is a binary variable: 1 = Household head received credit; 0 = Otherwise	+
TRAINING	Farmers training is a binary variable: 1 = Household head received training; 0 = Otherwise	+
GRPMBR	Group membership is a binary variable:1 = Farmer had group membership; 0 = Otherwise	+
LANDOWN	Land ownership is a binary variable: 1 = Ownership with a formal title deed; 0 = Otherwise	+
LABOUR	Access to timely labour is a binary variable: 1 = Farmer had access to labour; 0 = Otherwise	+
FAMIMPLNT	Access to animal-drawn farm implement is a binary variable: 1 = access to implement; 0 = Otherwise	+
RELIEF	Access to relief is a binary variable: 1 = Farmer received relief; 0 = Otherwise	+
SELLOUTPUT	Selling output is a binary variable: 1 = Farmer sold output; 0 = Otherwise	+
PERSOILFERT	Perception on soil fertility status is a dummy variable: 1 = Fertile; 0 = Otherwise	+

## 3 Results

### 3.1 Comparison of zai technology users and non-users in male-headed households and female-headed households

Among the interviewed households, (44%) female-headed households and (38%) male-headed households were utilizing zai technology (Table 3). On average, within female-headed households, the farming experience was significantly different at 10% level, with non-users of zai technology being more experienced in farming (21) when likened to users (17.28) (Table 3). The number of females in male-headed households varied significantly at 5% level, with user households having more females (2.93) as compared to non-users (2.53) (Table 3).

**Table 3:** Demographic factors influencing zai technology choice and use-intensity disaggregated by gender of the household head.

Variable	Female-headed households			Male-headed households		
	Mean			Mean		
	Non- users (n = 75)	Users (n = 58)	*T*-test	Non- users (n = 165)	Users (n = 102)	*T*-test
HH age	48.00	45.16	1.19	45.53	43.73	1.02
Farming experience	21.00	17.28	1.89^**c**^	18.78	17.71	0.68
Education	7.36	7.48	-0.16	8.41	9.10	-1.18
Household size						

*Number of males*	2.19	2.29	-0.41	2.75	2.81	-0.34
*Number of females*	2.71	2.79	-0.37	2.53	2.93	-2.16^**b**^

**b** and **c** represents 5% and 10% significance levels, respectively. HH represents Household Head.

There existed a significant relationship between group membership and choice and use-intensity of zai technology in male-headed households and female-headed households at the 1% level. About (53%) of zai technology users in female-headed households were members of a farmer group compared to (47%) non-users. For male-headed households, (46%) users had group membership compared to (54%) of non-users (Table 4). Within male-headed households, users and non-users also differed significantly at 1% in levels of participation in farmer training. About (51%) of users participated in farmer training, whereas (49%) non-users participated in training (Table 4). Also, results suggest a significant association at 1% level between choice and use-intensity of zai technology and access to extension services among male-headed households (Table 4).

**Table 4:** Socioeconomic factors influencing zai technology choice and use-intensity disaggregated by gender of the household head.

Variable	Female-headed households			Male-headed households		
	Non-users (n = 75)	Users (n = 58)	ꭓ^2^	Non-users (n = 165)	Users (n = 102)	ꭓ^2^
Off farm income	22 (49)	23 (51)	0.21	63 (59)	44 (41)	0.42
Sell output	64 (56)	50 (44)	0.88	138 (61)	87 (39)	0.72
Land ownership	51 (59)	35 (41)	0.36	107 (62)	67 (38)	0.90
Soil fertility perception						
*Fertile*	44 (57)	33 (43)	0.84	84 (61)	54 (39)	0.74
*Otherwise*	31 (55)	25 (45)		81 (63)	48 (37)	
Soil erosion severity						
*Not severe*	14 (64)	8 (36)	0.26	65 (64)	20 (36)	0.88
*Moderate*	59 (57)	45 (43)		115 (61)	74 (39)	
*Very severe*	2 (29)	5 (71)		15 (65)	8 (35)	
Participation in trainings	31 (51)	30 (49)	0.23	59 (49)	65 (51)	0.00 ^**a**^
Group membership	46 (47)	53 (53)	0.00^a^	95 (54)	80 (46)	0.00 ^**a**^
Group leadership	33 (46)	39 (54)	0.70	66 (57)	50 (43)	0.15
HH has received relief	14 (52)	13 (48)	0.59	35 (53)	31 (47)	0.09^**b**^
Received credit	19 (50)	19 (50)	0.35	44 (61)	28 (39)	0.89
Access to labour	63 (56)	49 (44)	0.94	138 (60)	92 (40)	0.13
Access to extension	23 (49)	24 (51)	0.20	31 (37)	52 (63)	0.00 ^**a**^
Farm implements	45 (52)	41 (48)	0.20	123 (62)	77 (38)	0.86
	Mean		*t*-test	mean		*t*-test
TLU	1.32	1.19	0.49	2.11	2.38	-0.67
Total land holding ha	1.59	1.63	-0.15	1.83	2.30	-2.04^**b**^
Total cultivated land ha	1.10	1.22	-0.99	1.26	1.75	-3.32 ^**a**^
Frequency of Trainings	1.03	0.97	0.30	0.62	1.28	-3.18 ^**a**^
Number of groups	0.75	1.26	-3.63^**a**^	0.76	1.13	-3.44 ^**a**^
Frequency of extension	0.45	0.67	-1.29	0.30	0.82	-4.37 ^**a**^
Market distance	56.00	63.02	-0.93	59.42	63.07	2.24

**a** and **b** represents 1% and 5% significance levels, respectively. % are in parentheses, HH represents household head, TLU represents Tropical Livestock Units.

Results also show a significant relationship at 5% level between access to relief and use of zai technology in male-headed households. Some (47%) non-users of zai technology had received government relief compared with (53%) users (Table 4). On average, total landholding significantly differed at 5% level within male-headed households, with users of zai technology having a larger land size compared with non-users (Table 4). Similarly, total cultivated land significantly differed within male-headed households at 1% level, with users of zai technology cultivating more land than non-users. Averagely, the frequency of training and extension contacts varied markedly for users and non-users of zai in male-headed households. Users of zai technology had more extension contacts and training as compared to non-users (Table 4).

### 3.2 Determinants of zai technology choice and use-intensity among male-headed households and female-headed households

Table 5 shows the Heckman 2-step model results of the determinants of zai technology choice and use-intensity. Findings revealed that, for both genders, land under cultivation significantly determined zai technology choice at the 5% level. A unit change in land under cultivation increased the likelihood of zai technology choice by 12.2% and 6.8% in female-headed households and male-headed households, respectively. Ownership and access to higher-value agricultural farm implement significantly influenced zai technology choice by women farmers at the 10% level. A unit change in access and ownership of higher-value agricultural farm implements increased the likelihood of choosing zai technology by 15.8% in female-headed households. Farmer group membership significantly influenced zai technology's choice in female-headed households and male-headed households at the 5% level. Specifically, group membership increased the likelihood of choosing zai technology by 26.8% and 8.8% among female-headed households and male-headed households, respectively. Within Male-headed households, an increase in agricultural extension access increased the likelihood of selecting zai technology by 5.9%.

**Table 5:** Estimated coefficient and the marginal effects of the Heckman 2-stage model on determinants of zai technology choice and use-intensity disaggregated by gender of the household head.

Variable	Pooled		Female-headed households		Male-headed households	
	Step I	Step II	Step I	Step II	Step I	Step II
	dy/dx	Coefficients	dy/dx	Coefficients	dy/dx	Coefficients
Age	-0.003 (0.002)	-0.011^**b**^ (0.005)	-0.005 (0.004)	-0.026 ^**a**^ (0.007)	-0.004 (0.003)	-0.019^**a**^ (0.006)
Education	-0.009 (0.007)	-0.042 ^**b**^ (0.016)	-0.009 (0.011)	-0.048^**c**^ (0.028)	-0.001 (0.006)	-0.003 (0.020)
Household size	-0.005 (0.010)	-0.003 (0.032)	-0.003 (0.019)	-0.014 (0.069)	0.007 (0.011)	0.012 (0.041)
Off farm income	0.029 (0.046)	0.120 (0.150)	0.057 (0.88)	0.285 (0.297)	0.005 (0.048)	0.019 (0.181)
Sell output	-0.024 (0.058)	-0.120 (0.184)	-0.047 (0.114)	-0.220 (0.371)	0.003 (0.071)	-0.067 (0.239)
Cultivated land (ha)	-0.028 (0.031)	-0.101 (0.099)	0.122^**b**^ (0.062)	0.596^**c**^ (0.207)	0.068^**b**^ (0.037)	0.361^**a**^ (0.107)
Land ownership	0.027 (0.047)	0.119 (0.154)	-0.053 (0.084)	-0.247 (0.287)	-0.004 (0.055)	0.009 (0.201)
Access to farm implements	-0.013 (0.051)	-0.061 (0.163)	0.158^**c**^ (0.093)	-0.742 (0.276)	-0.010 (0.061)	-0.065 (0.218)
Livestock densities	-0.005 (0.012)	-0.021 (0.037)	0.023 (0.025)	0.111^**a**^ (0.091)	-0.014 (0.012)	-0.052 (0.044)
Perception on soil fertility	-0.043 (0.047)	-	0.018 (0.074)	-	-0.016 (0.053)	-
Perception on soil erosion	0.055 (0.036)	0.209^**c**^ (0.125)	-0.030 (0.082)	-0.135 (0.250)	-0.035 (0.060)	0.250^**c**^ (0.159)
Farmer received training	0.086 (0.057)	0.349^**b**^ (0.180)	0.054 (0.093)	0.240 (0.359)	0.076 (0.062)	0.311 (0.230)
Group membership	0.142^**b**^ (0.072)	0.488^**b**^ (0.221)	0.268 ^**b**^ (0.111)	1.109 ^**a**^ (0.388)	0.088^**b**^ (0.087)	0.488 (0.221)
Received relief	0.127^**b**^ (0.061)	0.535^**a**^ (0.168)	-0.021 (0.086)	0.125 (0.342)	0.045 (0.058)	-0.208 (0.206)
Frequency of Trainings	-0.013 (0.017)	-0.056 (0.057)	0.027 (0.026)	0.140^**c**^ (0.081)	0.000 (0.017)	-0.003 (0.078)
Number of groups	0.019 (0.033)	0.083 (0.115)	-0.055 (0.050)	-0.291 (0.192)	-0.009 (0.040)	0.041 (0.153)
Access to agricultural credit	-0.033 (0.053)	-0.138 (0.163)	0.033 (0.082)	0.126 (0.318)	-0.032 (0.060)	0.189 (0.213)
Access to labour	0.074 (0.067)	0.324^**c**^ (0.191)	0.093 (0.111)	0.519 (0.335)	0.042 (0.072)	0.144 (0.254)
Distance to nearest market	0.000 (0.000)	0.001 (0.001)	0.000 (0.002)	0.002 (0.003)	0.000 (0.000)	0.001 (0.001)
Access to extension services	0.050^**c**^ (0.028)	-	0.022 (0.037)	-	0.059^**b**^ (0.037)	-
Statistic						
IMR(λ)	1.049 ^**b**^ (0.387)		0.989 ^**b**^ (0.499)		0.913 ^**b**^ (0.441)	
Number of observation	400		133		267	

a, b and c represents 1%, 5% and 10% significance levels, respectively. Standard errors are in parentheses.

Further, the results indicated that, age of the household head negatively and significantly (= -0.026, p < 0.01) and (= -0.019, p < 0.01) influenced zai technology use-intensity among female-headed households and male-headed households, respectively. Years of education negatively predicted zai technology use-intensity within female-headed households (= -0.048, p < 0.10). The study found a significant and positive relationship with zai technology use-intensity concerning livestock densities within female-headed households (= 0.111, p < 0.01). The coefficient of farmers' perception of soil erosion severity was significantly and positively associated with zai technology use-intensity among male-headed households (= 0.250, p < 0.10). The study also established a positive and significant relationship within female-headed households (= 0.140, p < 0.10) between the frequency of training on conservation practices and zai technology use-intensity.

## 4 Discussion

### 4.1 Demographic, socioeconomic, and farm characteristics of zai technology users and non-users

A majority of the interviewed households were male-headed. This finding collaborates with other studies conducted in the region by Mugwe et al. (2009) and Mwaura et al. (2021). The implication is that, men dominate major farm decision-making activities at the household level (Macharia et al., 2014). However, the results also suggest that more women farmers were using zai technology when compared to men. This probably explains the importance of women participating in agricultural decision making at household level and having access and control over productive resources such as land and income. The finding resonates with that of Murage et al. (2015), who reported that women adopted more climate-smart strategies when compared to men to avert the overarching constraints of climate shocks that affected them more directly than men. Our results further underscore the importance of larger households in driving choice and use-intensity of agricultural innovations. The propensity of choosing and using zai technology intensely was high in larger male-headed households. Usman et al. (2020) pointed out that larger families provide voluntarily available labour required in implementing labour-intensive technologies.

Farming experience has been found to positively as well as negatively influence the likelihood of adopting agricultural technologies (Knowler and Bradshaw, 2007). This could be associated with trade-offs involved in technology choice. With time, as farmers gain more experience, they gradually shift from technologies with diminishing marginal returns to improved technologies. Further, with rapid technological advancement, experience devalues with time necessitating frequent refreshment of knowledge for effectual technology choice and implementation decisions. This study's demographic characteristics show that, among female-headed households, non-users of zai technology were more experienced than users. Previous research by Ainembabazi et al. (2014) reported that farming experience is mostly important at the try-out stage. Then, farmers may opt out when the returns to investment start decreasing. More so, farmers may abandon zai technology that is labour intensive and requires more land allocation for intensive application.

Results showed that users of zai technology were members of farmer groups, accessed extension services, and participated in soil and water conservation training. These findings may be co-attributed to farmer groups, extension services, and training, providing capacity-building avenues to disseminate information to farmers on agricultural innovations. As was noted by Genius et al. (2014), extension agents and farmer groups link-up researchers and farmers reducing transaction costs when disseminating new and improved technologies to a larger heterogeneous group of farmers. In addition, through extension training, model farmers extend knowledge to other farmers through farmer to farmer training. Farm size is an important factor in the utilization process of agricultural technologies. Application of scale-dependent technologies depends on land size (Feder et al., 1985). User male-households had larger land size. This implies that zai technology is a lumpy technology requiring large farm sizes to maximize returns on investment.

### 4.2 Determinants of zai technology choice among male-headed households and female-headed households

For both genders, membership in a farmer group increased the likelihood of choosing zai technology. Group membership and other social forums could provide linkage to access agricultural information through extension contacts and other farmers' interactions where they exchange ideas and practically demonstrate agricultural innovations. Also, farmer groups are target points for researchers' and other development agents disseminating research findings. These results are consistent with the finding of Gido et al. (2015) and Kassie et al. (2014), who reported that farmer groups and other rural institutions create avenues through which information on agricultural innovations is channelled to farmers reducing the cost of information delivery through increased economies of scale. Further, in a group platform, early adopters can share their testimonies (success stories), encouraging other members to adopt the practices (Mango et al., 2017).

Among male-headed households, access to extension increased the likelihood of choosing zai technology. Extension services bridge farmers’ knowledge gaps on improved farming practices and application modalities. Our finding was in agreement with several other studies, for example, Gebregziabher (2018), Donkor et al. (2019) and Ehiakpor et al. (2019). These studies noted that extension contact increased smallholder farmers' probability of adopting zai technology, among other soil conservation technologies. Additionally, the results are consistent with those of Mponela et al. (2016), who found extension services to positively determined the choice of soil conservation practices. Also, Ndiritu et al. (2014) found that the probability of adopting chemical fertilizer increased with access to agricultural extension. Conversely, Chirwa et al. (2008) reported that extension contacts may sometimes not result in increased technology use. This may arise when extension agents have preferential approaches targeting resource-poor households who lack resources necessary for implementing new technologies.

Within male-headed and female-headed households, total land cultivated positively influenced the choice of zai technology. This was an indication that larger farm sizes increased the likelihood of choosing zai technology. This could be attributed to flexibility of devoting a portion of land for new technologies increasing with increase in land size. Our results corroborate with the findings of Kassie et al. (2010), Mwangi and Kariuki (2015) and Gebre et al. (2019), who found that increasing land size under cultivation increases the likelihood of utilizing agrarian technologies among smallholder households with an explanation that, the land is an indicator of wealth, which relaxes capital constraints of implementing the practices. Contrariwise, Thinda et al. (2020) contend that large farms are not always a prerequisite for the choice of agrarian technologies. Farmers' with large farm size may prioritize labour-saving technologies abandoning labour-intensive technologies such as zai pits. In addition, farmers may fail to adopt zai technology as it hinders animal traction, a cheaper alternative source of farm power when compared with other ploughing mechanization for resource poor households (Kimaru-Muchai et al., 2020).

Ownership and access to farm implements (a proxy of household wealth in productive assets) within female-headed households increased the likelihood of choosing zai technology. This could be due to the availability of farm implements, which save on both time and labour costs for women farmers trapped in drudgery rural agriculture. This agrees with Johnson et al. (2016), who reported that household assets could influence the use of agricultural interventions among women farmers and increase returns to productive assets. The study also notes that farmers with low-value farm assets are limited to low-impact technologies that are appropriate with low-value agricultural implements. Similarly, Peterman et al. (2014) reported that farm implements significantly determined the choice of agricultural technologies for men and women farmers.

In female-headed households, training on soil and water conservation increased the likelihood to use zai technology intensely. Training increases farmers’ knowledge on agricultural technologies application modalities. Additionally, frequent knowledge-refreshing increase the chances of continued adoption after try-out stage. Well-versed farmers make accurate estimates of expected returns, a cushion from frustrations of returns overestimation resulting in stagnating and abandoning technologies. The results are consistent with Li et al. (2020) who found information accumulation to have a positive and significant impact on technology adoption. In another study by Okeyo et al. (2020) also found that farmer training positively influenced improved sorghum varieties' adoption among smallholders. Further, he reported that, trained farmers are better informed on varying production patterns under changing agroclimatic conditions and often they prefer climate smart agriculture. Moreover, a study by Gebre et al. (2019) reported that participation in farmer training had more effect on increasing farmers' ability to effectively apply of new technologies.

Kimaru-Muchai et al. (2020) pointed out that the probability of zai technology use is higher among younger farmers. The study attributed the finding to the labour-demanding nature of zai technology and younger farmers having a better understanding and up-to-date information on zai technology application modalities. In agreement with our current finding, Asfaw and Neka (2017) reported that age negatively influenced acceptance level and use of conservation practices. The negative interaction between age and use of the practices was ascribed to age decreasing farmer assertiveness, hence reducing farm care involvement. Contrary, Wekesa et al. (2018) noted that, farming experience increases with age and farmers upgrade from smaller agrarian practices packages to more rewarding options.

We established a negative relationship between years of education and zai technology use-intensity, suggesting that more educated farmers were more inclined towards non-farming activities. The findings were consistent with those of Alwang et al. (2019) and Okeyo et al. (2020), who reported that educated farmers are more knowledgeable in predicting and analysing agricultural-related risks and uncertainties associated with biophysical and agro-ecological conditions. In addition, educated farmers may opt-out from farming, taking up secondary non-farming opportunities that are better rewarding, secure, and offer a wide range of alternatives. However, this finding is inconsistent with Mango et al. (2017) and Wordofa et al. (2020), who found the education level of the household head to influence the choice of soil and water conservation practices positively. These studies attributed their findings to the influence of education in raising farmer receptiveness on important conservation measures.

Male-headed households who perceived soil erosion to be severe were more likely to use zai technology intensely. An implication is that, zai technology has the water-holding capacity and when applied together with manure, soil water infiltration and porosity improve and, subsequently, reduces water loss. Low soil fertility occurs as a result of soil loss, among other factors; hence farmers who experience soil loss adopt zai technology more (Kimaru-Muchai et al., 2020). A study by Biratu and Asmamaw (2016) points out that farmers who perceived soil erosion on their farmland as a problem and had good motives to implement soil water conservation activities.

In terms of total livestock densities, we found a positive relationship with zai technology use-intensity within female-headed households. Livestock ownership signifies women's empowerment in agriculture, translating to household wellbeing (Kristjanson et al., 2014). Proceeds from livestock can be ploughed back to cater to costs of labour-demanding zai technology. Most commonly, zai pits are applied in combination with animal manure; hence households with readily available animal manure are more likely to allocate more land under zai technology. These findings agree with Ndiritu et al. (2014), who found livestock ownership to influence soil conservation measures positively. Further, their study pointed out that female plot managers faced with resource constraints (livestock, credit, and labour) have reduced chances to use soil conservation measures compared with male plot managers.

## 5 Conclusion

The study supports the hypothesis that there are gender specific determinants of zai technology choice and use-intensity in Upper Eastern Kenya's drylands. In particular, within male-headed households, we found that efforts to promote zai technology should consider the total cultivated land, farmers' perceptions on soil erosion, group membership, and access to extension services. For female-headed households, total land cultivated, livestock densities, group membership, and frequency of training and ownership and access to farm implements were important determinants of zai technology choice and use-intensity. Livestock ownership, access to land, and farm implements are proxy measures for women's empowerment in agriculture, driving utilization of agricultural innovations intensely. This calls for the need to develop gender-sensitive policies that advocate equitable and secure ownership of productive assets. Such policy frameworks could be embraced as a guideline to women's empowerment in agriculture. Moreover, the study recommends that extension systems need to be reformed and tailored to serve men and women farmers' specific needs and preferences with regard to utilization of agricultural innovations. This will enable both women and men farmers, to choose and use zai technology as an adaptation strategy to climate shocks in sub-Saharan Africa's drylands.

## Declarations

### Author contribution statement

Amos Mwenda Ndeke: Conceived and designed the experiments; Performed the experiments; Analyzed and interpreted the data; Wrote the paper.

Jayne Njeri Mugwe; Hezron Mogaka; George Nyabuga: Conceived and designed the experiments; Wrote the paper.

Milka Kiboi; Felix Ngetich; Monicah Mucheru-Muna; Isaya Sijali; Daniel Mugendi: Contributed reagents, materials, analysis tools or data; Wrote the paper.

### Funding statement

This work was supported by the Flemish Interuniversity Council-University Development Co-operation through the (VLIR-UOS Project "Climate-Smart Options Allowing Agricultural Intensification Among Smallholder Farmers in the Dry Zones of the Central Highlands of Kenya").

### Data availability statement

Data will be made available on request.

### Declaration of interests statement

The authors declare no conflict of interest.

### Additional information

No additional information is available for this paper.

## Appendix A Supplementary data

Supplementary Materials 6 2 21_V2 Supplementary Materials 6 2 21_V2
