# Supplementary material for: Gender-specific determinants of Zai technology use intensity for improved soil water management in the drylands of Upper Eastern Kenya
Source: Heliyon. 2021 Jun 4;7(6):e07217. doi: 10.1016/j.heliyon.2021.e07217 (PMC8207220; doi:10.1016/j.heliyon.2021.e07217)
Supplement: Supplementary Materials 6 2 21_V2 [file mmc1.docx]

**Supplementary Materials**

**Appendix 1: Questionnaire**

**Questionnaire No……………**

You are among several farmers in this area who have been selected for this study. The study seeks to evaluate the influence of gender on use of selected soil fertility and soil water conservation strategies. The information provided will be treated with utmost confidentiality. Your assistance in answering the questions truthfully and accurately will be highly appreciated.

Date …………………. Enumerator’s name………………………………….

**Geographical location**

Ward ………………………….

**Farmer characteristics**

1. Gender of the household head 1=Male 0 =Female

2. Gender of household decision maker *(Who makes key agricultural decisions at household level i.e. relate to household headship)* 1=Male 0 =Female

*Follow-up on gender of the household decision maker*

| ***Major decision making activity at household level*** | ***Decision maker (indicate the decision maker i.e. 1 or 0*** | ***Remarks*** |
| --- | --- | --- |
| *What crops to grow* |  |  |
| *What size of land to cultivate* |  |  |
| *Which SWC technologies to use* |  |  |
| *Allocation of agricultural credit* |  |  |
| *Control of farm income* |  |  |
| *Farm produce sales* |  |  |
| *Labour allocation* |  |  |

3. Age of the household head …………………. years

4. Years of education of household head ……...years

*What is your education level? 1=Primary, 2= Secondary, 3=Tertiary 4=Non formal education*

5. Farming experience of the household head (*years the farmer has been making key farming decisions independently*) …….

6. Household size

*Number of males ………*

*Number of females………*

7. Does the household head participate in off-farm employment? 1= Yes 2=No

*If yes, what is the average monthly income from off-farm employment………. Ksh*

8. Are you a member of farmer based group/ organisation? 1= Yes 2=No

*If yes, how many groups/ organisations ………….*

*If yes, what benefits does he/she derive from group membership?*

*1=Information on credit 2=General advice on farming 3=Information on climate change 4=Help in credit access 5=Others (specify)……*

**Farm characteristics**

9. Do you own land that you are currently farming on with a formal title deed? 1=Yes 2=No

*What is the size of land owned?...............acres*

10. What is the total size of land under cultivation? …………...acres

11. Have you sold farm produce in the last one cropping season? 1=Yes 2=No

12. Do you have access to timely labour? (*during planting, harvesting periods or when applying SWC measures*) 1=Yes 2=No

13. Do you have access to animal drawn farm equipment? *(“access” includes both ownership and renting)* 1=Yes 2=No

14. Do you keep livestock? 1=Yes 2=No

*If yes, which livestock and how many of each do you keep in your farm? Please tick on livestock owned and indicate the number*

| ***Type of livestock*** | ***Number*** |
| --- | --- |
| *1=Cattle* |  |
| *2=Sheep* |  |
| *3=Goats* |  |
| *4=Donkeys* |  |
| *5=Chicken* |  |
| *6=Pigs* |  |
| *7=Any other specify ………………………….* |  |

15. Have you received any agricultural extension services within the last one year? 1=Yes 2=No

16. Have you received training on soil fertility and soil water management within the last one year? 1=Yes 2=No

*If yes, how many times were you trained in the last one cropping season…………….*

17. Do you have access to agricultural credit? 1=Yes 0=No

*If yes, indicate the amount received last year……………. Ksh*

*Is the credit used in improving soil fertility and soil water management? 1=Yes 0=No*

18. What is the distance in walking minutes to the nearest input/output market?...........

19. Have you received any form of relief either in form of inputs, farming implements or cash? *1=Yes 0=No*

*If yes, was the relief beneficial in addressing soil infertility and soil water stress? 1=Yes 0=No*

**Soil water conservation**

20. Are you aware of zai technology/pits? *(try to interrogate the respondent with more questions on measurements, spacing and application modalities to clearly know if they are aware)* 1= Yes 0=No

21. Are you using or have used zai technology in the last one cropping season? *(Where applicable, make observations*)1= Yes 0=No

*If yes, what is the size of land is under zai technology? (confirm the size of land by measuring) ........acres*

*If yes, when incorporating zai do you add manure?* 1= Yes 0=No

*If yes, when incorporating zai do you add mineral fertilizer?* 1= Yes 0=No

22. What is the terrain of the cultivated land? 1=Sloppy 0=Otherwise

23. How do you perceive soil fertility status of the cultivated farm? 1=Very fertile, 2= moderate 3=Poor

24. Have you experienced soil erosion in your farm? 1= Yes 0=No

*If yes, how do you perceive the severity of the soil erosion in your farm currently? 1=Very severe 2= Moderate 3= Not severe*

***Thank you***

**Appendix 2: Description of independent Variables**

HHAGE: the age of household head was measured in years. Studies have found that the probability of using soil and water conservation practices intensely is higher among younger farmers as compared to ageing farmers (Asfaw & Neka, 2017). Conversely, other studies have found age to positively influence choice of soil management practices. Therefore, the study hypothesized a negative relationship between age and Zai technology choice and use intensity.

HHSIZE: household size refers to the number of people who live in a household. An important factor influencing use of labour-intensive agricultural technologies. Larger households have more human capital in terms of labour to adopt labour demanding soil and water conservation technologies (Belachew et al., 2020). The study hypothesized household size to positively influence the likelihood of choosing and using zai technology intensely.

HHEDUC: education of the household head refers to years of schooling. Education has been found to positively and negatively influence utilization of agrarian innovations. Educated farmers are presumed to be more informed on soil water conservation technologies application modalities and hence being more receptive to new and improved technologies (Mango et al., 2017). Conversely, educated farmers may opt out from farming for off-farm activities that are more rewarding (Alwang et al., 2019). Our study hypothesized a positive/negative relationship between education and zai technology choice and use-intensity.

EXTENSION: access to extension services measured the availability of extension services to farmers. This refers to contact of development/extension agents with farmers within a period of one year. Extension services play a great role in creating awareness on soil and water conservation technologies (Mponela et al., 2016). Therefore, access to extension was hypothesized to positively and significantly influence zai technology choice and use-intensity.

LIVSTCK: the total livestock densities is an essential factor in explaining adoption of agricultural technologies at household level (Ndiritu et al., 2014). We hypothesized livestock densities to positively influence zai technology choice and use-intensity positively.

PERCSOILERSN: perceiving soil erosion to be more severe has been found to positively influence the likelihood of using soil and water conservation technologies. Severe soil erosion increases nutrient and surface water loss (Biratu & Asmamaw, 2016). We expected that perception on soil erosion to positively influence zai technology choice and use-intensity.

FAMEXP: the years a farmer has been involved in making key farming decisions has been found to have mixed effects on choice and use-intensity of agricultural innovations. More experienced farmers are reputed to have a better knowledge of conservation agriculture hence a higher likelihood of adopting soil and water conservation technologies (Knowler & Bradshaw, 2007). The study hypothesized a positive relationship between farming experience and zai technology choice and use-intensity.

MKTDST. distance in walking minutes to the nearest input/output market was hypothesized to negatively influence zai technology's choice and use-intensity (Muriithi et al., 2018; Wekesa et al., 2018).

LAND: total cultivated land is often correlated with farm income and wealth. Therefore, farmers with large farm size could have the ability to pay wages while undertaking soil and water conservation. Additionally, farmers with large farm size have the flexibility of experimenting with new technologies (Thinda et al., 2020). The study hypothesized a positive association between land and zai technology choice and use-intensity.

CREDIT: access to agricultural credit promotes the adoption of labour-intensive climate-smart innovations, among other technologies, that require high initial investment capital (Obisesan et al., 2016; Yigezu et al., 2018). Access to agricultural credit was hypothesized to play a positively effect zai technology choice and use-intensity

TRAINING: participating in farmer training is a dummy variable 1 for trained household heads and 0 otherwise. We hypothesized that training increases farmers' knowledge of soil and water conservation technologies, hence positively influencing zai technology's choice and use-intensity (Okeyo et al., 2020).

GRPMBR: group membership increases the likelihood of adopting agricultural innovations (Kassie et al., 2014). Besides, agricultural development agencies who work closely with farmers' groups have higher success rates. The study also hypothesized that membership to farmer groups/organizations positively influenced choice and use-intensity of zai technology.

LANDOWN: measures if a farmer-owned land with a formal title deed or not. A secure land tenure system increases the likelihood of farmers adopting long-term agricultural innovations (Awotide et al., 2016). The study hypothesized a positive correlation between land ownership with a formal title deed and choice of zai technology.

LABOUR: measures if a farmer had access to timely labour. Farmers with access and the ability to hire farm hands have a higher probability of adopting soil water management innovations, among other agricultural technologies that are labour demanding (Mugwe et al., 2009; Teshome et al., 2016). Access to timely labour is therefore expected to positively influence the choice and use-intensity of zai technology positively.

FAMIMPLNT: technology adoption is positively influenced by access to and ownership of farm implements (Melesse, 2018). The study hypothesized access to farm implement to play a significant role in choice and use-intensity of zai technology.

RELIEF: farmers receiving relief from the government and other development agencies act as incentives to technology adoption. Awotide et al. (2016) found that farmers who had received relief in the form of improved seeds commercialized rice production in Nigeria. We hypothesized a positive relationship between farmers receiving relief in the form of inputs and utilization of zai technology.

SELLOUTPUT: selling farm output is a proxy of farm income. Availability of farm income positively predicts choice and use-intensity of soil and water conservation technologies (Kuehne et al., 2017). The study hypothesized a positive relationship between the sale of output and utilization of Zai technology. Also, it was expected that off-farm income would have the same effect.

PERSOILFERT: measures farmer's perception of soil fertility status at farm level. Farmers who perceive their soils as infertile are more likely to use conservation practices (Belachew et al., 2020). Assuming a farmer has the ability to implement zai technology, it is expected perception that soils are infertile increases the likelihood of choosing zai technology and allocate more cultivated land under the technology with regard to its ability to improve soil fertility and soil water conservation.

REFERENCES

Awotide, B. A., Karimov, A. A., & Diagne, A. (2016). Agricultural technology adoption, commercialization and smallholder rice farmers' welfare in rural Nigeria. *Agricultural and Food Economics*, *4*(1), 3.

Kuehne, G., Llewellyn, R., Pannell, D. J., Wilkinson, R., Dolling, P., Ouzman, J., & Ewing, M. (2017). Predicting farmer uptake of new agricultural practices: A tool for research, extension and policy. *Agricultural Systems*, *156*, 115–125.

Melesse, B. (2018). A review on factors affecting adoption of agricultural new technologies in Ethiopia. *Journal of Agricultural Science and Food Research*, *9*(3), 1–4.

Muriithi, B. W., Menale, K., Diiro, G., & Muricho, G. (2018). Does gender matter in the adoption of push-pull pest management and other sustainable agricultural practices? Evidence from Western Kenya. *Food Security*, *10*(2), 253–272.

Obisesan, A. A., Amos, T. T., & Akinlade, R. J. (2016). Causal Effect of Credit and Technology Adoption on Farm Output and Income: The Case of Cassava Farmers in Southwest Nigeria. *5th International Conference of the African Association of Agricultural Economists*, (No. 310-2016-5370).

Teshome, A., de Graaff, J., & Kassie, M. (2016). Household-level determinants of soil and water conservation adoption phases: Evidence from North-Western Ethiopian highlands. *Environmental Management*, *57*(3), 620–636.

Yigezu, Y. A., Mugera, A., El-Shater, T., Aw-Hassan, A., Piggin, C., Haddad, A., Loss, S. (2018). Enhancing adoption of agricultural technologies requiring high initial investment among smallholders. *Technological Forecasting and Social Change*, *134*, 199–206.
